# Mouse models of Alzheimer’s disease cause rarefaction of pial collaterals and increased severity of ischemic stroke

**DOI:** 10.1007/s10456-018-9655-0

**Published:** 2018-12-05

**Authors:** Hua Zhang, Bo Jin, James E. Faber

**Affiliations:** 1 Department of Cell Biology and Physiology, University of North Carolina at Chapel Hill, 6309 MBRB, Chapel Hill, NC 27599-7545 USA; 2 McAllister Heart Institute, University of North Carolina at Chapel Hill, Chapel Hill, USA; 3 Zhejiang Chinese Medical University, Hangzhou, China

**Keywords:** Collateral circulation, Alzheimer’s disease, Cerebral amyloid angiopathy, Stroke

## Abstract

Vascular dysfunction contributes to the progression and severity of Alzheimer’s disease (AD). Patients with AD also sustain larger infarctions after ischemic stroke; however, the responsible mechanisms are unknown. Pial collaterals are the primary source of protection in stroke. Unfortunately, natural aging and other vascular risk factors cause a decline in collateral number and diameter (rarefaction) and an increase in stroke severity. Herein, we tested the hypothesis that AD accelerates age-induced collateral rarefaction and examined potential underlying mechanisms. Triple and double transgenic mouse models of AD both sustained collateral rarefaction by 8 months of age, well before the onset of rarefaction caused by aging alone (16 months of age). Rarefaction, which did not progress further at 18 months of age, was accompanied by a twofold increase in infarct volume after MCA occlusion. AD did not induce rarefaction of similarly sized pial arterioles or penetrating arterioles. Rarefaction was minimal and occurred only at 18 months of age in a parenchymal vascular amyloid-beta model of AD. Rarefaction was not associated with amyloid-beta deposition on collaterals or pial arteries, nor was plaque burden or CD11b^+^ cell density greater in brain underlying the collateral zones versus elsewhere. However, rarefaction was accompanied by increased markers of oxidative stress, inflammation, and aging of collateral endothelial and mural cells. Moreover, rarefaction was lessened by deletion of CX_3_CR1 and prevented by overexpression of eNOS. These findings demonstrate that mouse models of AD promote rarefaction of pial collaterals and implicate inflammation-induced accelerated aging of collateral wall cells. Strategies that reduce vascular inflammation and/or increase nitric oxide may preserve collateral function.

**Electronic supplementary material:**

The online version of this article (10.1007/s10456-018-9655-0) contains supplementary material, which is available to authorized users.

## Introduction

Alzheimer’s disease (AD) affects more than 45 million people and is increasing with aging of the world’s population [1]. The familial form is caused by mutations of amyloid precursor protein (APP) and γ-secretases, presenilin-1 and -2, while alleles of *APOE, TREM2*, and several other genes, along with risk factors such as aging, diabetes, obesity, and hypertension, increase the risk of developing the more common sporadic late-onset form of the disease [2–5]. According to the “amyloid hypothesis”, genetic and environmental factors result in altered processing of APP, accumulation of intracellular amyloid-beta peptides (Aβ) and the microtubule protein tau, imbalance between production, degradation, and clearance of Aβ leading to accumulation of extracellular Aβ, activation of microglia and astrocytes, and a cascade of events resulting in inflammation, synapse dysfunction, neuronal loss, and cognitive decline [2–5].

There is also substantial evidence that vascular abnormalities and the above neurodegenerative processes interact synergistically early-on to increase the risk and progression of AD [6–10]. Patients and animal models of AD undergo accumulation of Aβ on blood vessels (cerebral amyloid angiopathy, CAA), recruitment of peripheral immune cells to the perivascular niche, disturbances in smooth muscle cell (SMC) reactivity and regulation of blood flow, and altered blood–brain barrier integrity. As well, increased risk of acute ischemic stroke, larger lesions, and worse outcomes have been reported in clinical and experimental studies of AD. Presence of *APOE-ε4* allele and CAA on autopsy increased the odds of cortical infarction threefold [11]. Other studies have also found, although not universally [12, 13], that AD increases the risk of ischemic stroke [14]. In mouse models of AD, penumbral blood flow was lower and infarct volume larger following permanent MCA occlusion (pMCAO) [15–19] and photothrombotic occlusion [20]. The mechanisms responsible for these findings in stroke have not been investigated.

Pial collaterals are the primary source of protection in acute ischemic stroke [21–24]. Thus, AD-induced insufficiency of the pial collateral circulation could contribute significantly to the above findings. Conductance of the collateral network is mainly determined by the number and average diameter (i.e., extent) of pial collaterals interconnecting the affected territory to adjacent arterial trees [23, 24]. Unfortunately, collateral extent varies widely among individuals. In mice, pial collaterals form late during gestation and undergo perinatal maturation that determines their extent in the adult [25–28]. This collaterogenesis process varies widely due to differences in genetic background [23, 24, 26]. Environmental factors also affect collateral extent. Aging, hypertension, diabetes, endothelial dysfunction, and other vascular risk factors cause a decline or pruning away of collaterals and a smaller lumen diameter in those that remain (collateral rarefaction) [29–32]. Indirect measures of collaterals assessed during the hyperacute stage of stroke indicate that collateral conductance also varies widely in humans and is adversely affected by vascular risk factors [21, 22, 33–35]. The cause of this risk factor-induced rarefaction of collaterals, which does not occur in other pial vessels [29, 31], has been suggested to extend from a unique sensitivity of collaterals, created by the low and disturbed shear stress and high wall stress normally present in them, to diseases and conditions that increase oxidative stress and inflammation [29–32]. Of note, traditional vascular risk factors also increase the risk of, or are strongly associated with, AD and vascular cognitive impairment [3, 7, 36–39].

The purpose of this study was to determine if collateral rarefaction and more severe acute ischemic stroke occur in mice with AD and explore possible mechanisms. We studied single (*APP*SwDI), double (*APP*695, *PSEN-1*), and triple (*APP*Sw, *PSEN-1, MAPT* (Tau)) transgenic mouse models of AD at 1, 8, and 18 months of age.

## Methods

See the Online Supplement for details. Triple transgenic AD mice (3xTg-AD) were C57BL/6J (B6).129-*PSen1*^*tm1Mpm*^ Tg (APPSwe, tauP301L)1Lfa/Mmjax (homozygous) [40]; wildtype (WT) controls were B6; 129S-F1/2J. 2xTg-AD mice were B6.Cg-Tg(APP695)3Dbo Tg(PSEN1dE9) S9Dbo/Mmjax] (hemizygous) [41]; controls were B6 WT littermates. 1xTg-AD mice were B6.Tg(Thy1-APPSwDutIowa) BWevn/Mmjax] (homozygous) [42]; controls were B6/J.B6/NJ-F1. B6.CX_3_CR_1_^−/−^ were B6.129P(Cg)-Ptprc^a^ Cx3cr1^tm1Litt^/LittJ. B6.eNOS transgenic mice have a twofold increase in NO production [32]. Approximately, equal numbers of males and females were studied in each experiment. The scope of this study did not include the investigation of the effects of sexual dimorphism in AD-induced collateral rarefaction. In a previous study, the observed effects of sex were negligible for native pial collateral number, diameter, and amount of rarefaction seen with aging in 3.5-, 8-, and 22-month-old B6 mice or 8 versus 18-month-old B6; 129SvEv mice [43]. Angiography was performed after perfusion-fixation at maximal dilation and filling of the pre-capillary vessels with viscosity-adjusted Microfil^®^ [32]. All pial collaterals between the ACA and MCA trees of both hemispheres were identified and their anatomic (maximally dilated) lumen diameters were measured at midpoint and averaged for each animal. Collateral length, tortuosity, calculated relative resistance of the MCA-to-ACA collateral network, territories of the ACA, MCA, and PCA trees, and other morphometrics were also measured [23, 32]. Number and average diameter of the penetrating arterioles (PAs) diverging from the collaterals and branches of the ACA tree overlying the dorsal cortex were measured after optical clearing. Permanent MCA occlusion (pMCAO) was by occlusion of the M1-MCA just distal to the lenticulostriate branches [32]. Infarct volume 24 h later was by 2,3,5-triphenyltetrazolium chloride, and immunofluorescent histology was by standard techniques [32]. The STAIR criteria adhered to included blinding to groups before and during analysis and no exclusion of data points.

## Results

### Overexpression of mutant APP and presenilin-1 causes rarefaction of pial collaterals

**Fig. 1:**
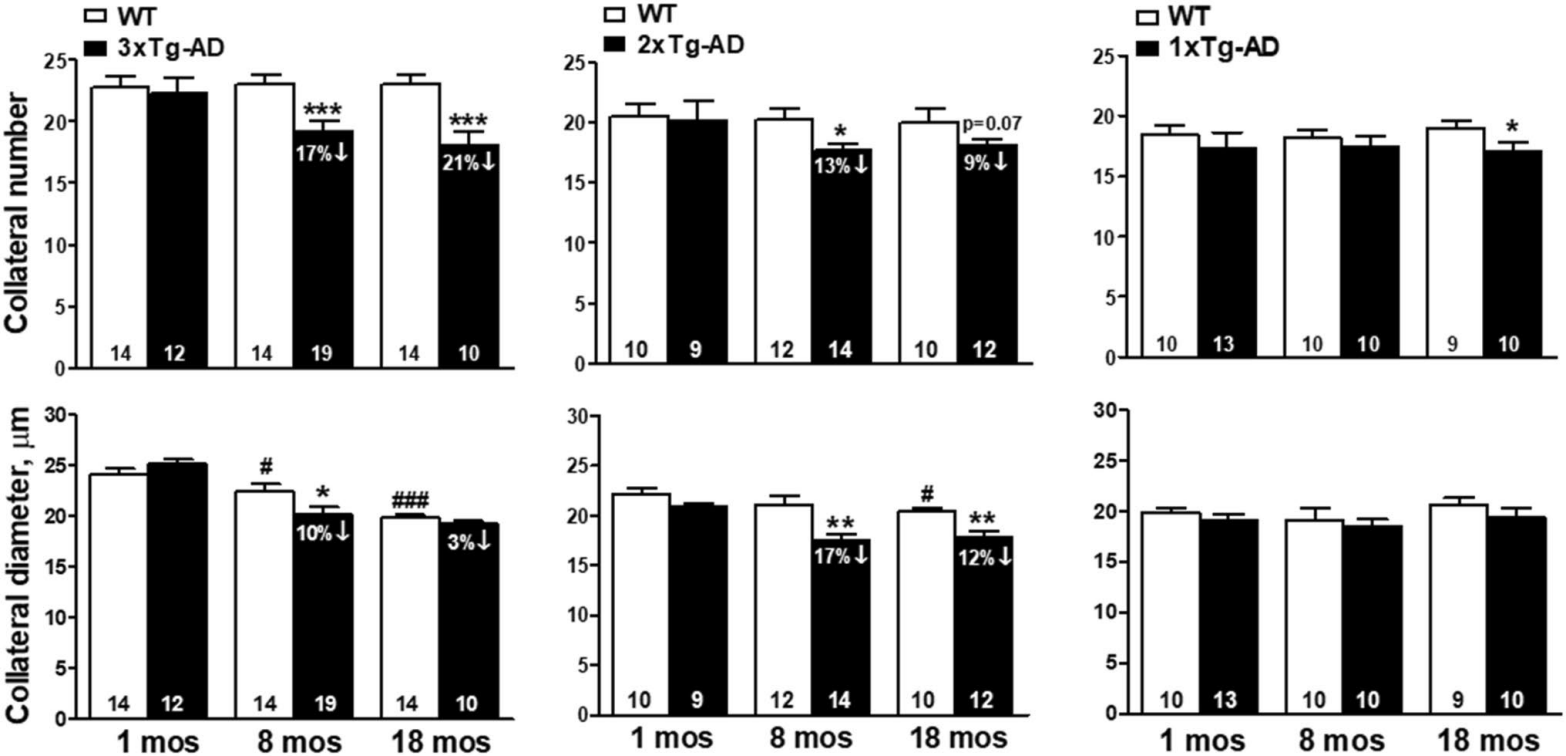
Overexpression of mutant APP and presenilin-1 causes rarefaction of pial collaterals. Rarefaction in 3xTg-AD and 2xTg-AD mice, i.e., decline in collateral number and lumen diameter, occurred at an earlier age (mos, months) than rarefaction caused by aging alone. The latter was observed for diameter at 16 months of age and number at 24 months of age, respectively [36], a finding confirmed in 18-month-old 3xTg and 2x-Tg wildtype (WT) controls. 1xTg-AD mice evidenced a small loss in number at 18 months of age. In this and subsequent figures and tables, unless indicated otherwise: the total number and average lumen diameter of all MCA–ACA collaterals of both hemispheres are shown, values are mean ± SEM, and numbers of animals are given in the bases of the bars. *^,^**^,^***p < 0.05, 0.01, 0.001 versus age-matched WT. ^#,###^*p* < 0.05, 0.001 1-month-old WT

Collateral number and diameter were reduced by similar amounts in 8-month-old 3xTg- and 2xTg-AD cohorts (~ 28 human-year equivalents, hye [44]), compared to cohorts of controls comparable in age (Fig. 1). No additional decrease occurred at 18 months of age (~ 56 hye). Absence of effect at 1 month indicates that the presence of fewer and smaller collaterals is not due to decreased collaterogenesis, which occurs in the perinatal period and determines collateral number and diameter in the adult [25–28]. The latter could possibly have occurred secondary to parenchymal Aβ that has been detected in 1-month-old transgenic AD mice [45], or from non-specific effects of transgene insertion or private mutations/genetic drift in the AD strains. Rarefaction in the 8-month-old AD mice occurred much earlier than that caused by aging alone. The latter, which becomes evident at 16 and 24 months of age as a decrease in diameter and number, respectively, in B6 mice [29], was confirmed for diameter in 3xTg and 2x-Tg WT mice at 18 months of age (Fig. 1). Although difficult to detect, occasional collaterals were observed, presumably late in the process of being pruned away, as a narrowing of the lumen of the center-most segment of a collateral to that approximating nearby capillaries (Supplemental Fig. I). 1xTg mice sustained no rarefaction at 8 months of age, and only a small decrease in collateral number was evident at 18 months of age (Fig. 1). This difference may reflect that, unlike in the 2xTg and 3xTg strains, vascular pathology in the 1xTg model manifests as deposition of Aβ on intracerebral capillaries and arterioles with little deposition on pial vessels [42, 46, 47].

**Fig. 2:**
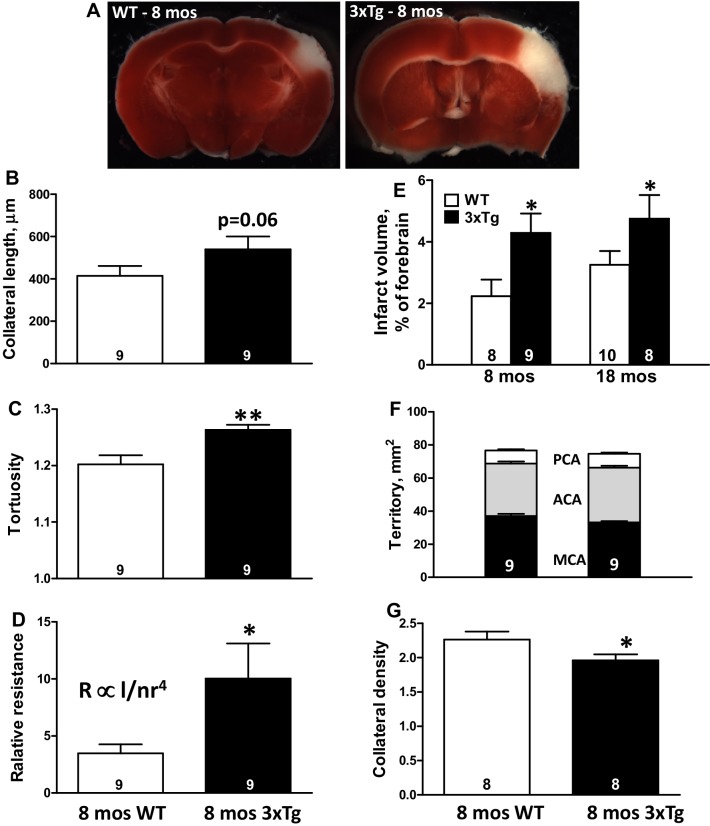
AD-induced collateral rarefaction is accompanied by increased collateral tortuosity, resistance of the collateral network, and infarct volume (**a**–**e**), and is specific for collaterals. **a** Representative images of infarct volume (unstained/white). **d** Calculated relative resistance of the collateral network [average collateral length ÷ (collateral number × average collateral radius [4])]. Increased infarct volume was also seen in 18-month-old 3xTg mice. The latter was in association with modestly reduced brain and body weight and area of the collateral zone between the MCA–ACA trees (Supplemental Fig. II). **f** Territories of the cerebral artery trees. Collateral density (number per mm^2^ of collateral zone) remained reduced when normalized to collateral zone area (**g**). No loss of number or diameter occurred for distal-most arterioles of the MCA tree, which are nearby and of similar diameter as collaterals (Supplemental Fig. II), or for penetrating arterioles branching from the extant collaterals (Supplemental Fig. III). *^,^**p < 0.05, 0.01 versus WT

We quantified additional parameters in a second group of 8-month-old 3xTg and WT mice (Fig. 2). Collateral length, tortuosity, and calculated relative resistance of the collateral network interconnecting the MCA and ACA trees were increased in the AD mice by 37%, 34%, and 2.9-fold, respectively (Fig. 2a–d). Infarct volume 24 h after pMCAO was increased 91% (Fig. 2e), even though MCA tree territory tended to be slightly smaller (Fig. 2f). Infarct volume increased less (46%) in 18-month-old 3xTg mice, reflecting the larger lesion evident in the age-matched WT controls (Fig. 2e). Body weight, brain weight, and area of the collateral zone between the MCA and ACA trees were modestly lower (Supplemental Fig. II). The latter is consistent with the smaller brain weight, which also occurs in human familial and sporadic AD [48]. Collateral number, when normalized to area of the collateral zone, remained reduced (Fig. 2g), indicating that collateral rarefaction was not secondary to the modest brain atrophy. No decrease in number and diameter occurred for distal-most arterioles (DMAs) of the pial artery trees, which have diameters similar to collaterals, or for PAs branching from the pial arteries/arterioles and extant collaterals in the cohort strains examined (Supplemental Figs. II and III). The above findings indicate that collaterals undergo selective rarefaction at an early age in AD.

### Rarefaction does not require aggregation of Aβ on collaterals

**Fig. 3:**
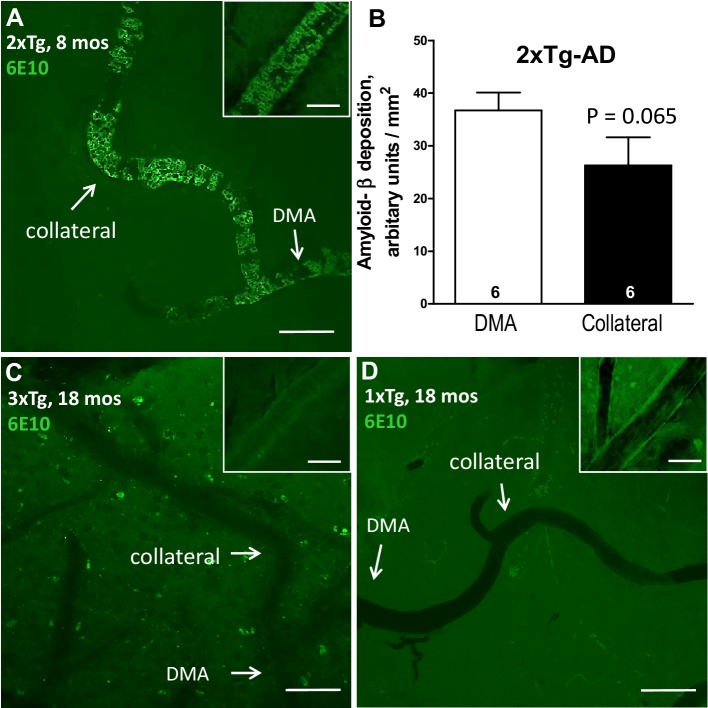
Rarefaction does not require deposition of Aβ on collaterals. Images and data analysis were obtained from whole-mounts of the dorsal surface of the cortex. Rarefaction in 3xTg and 2xTg mice (Fig. 1) does not correlate with deposition of beta-amyloid on collaterals or upstream arterioles and arteries. 2xTg mice have strong deposition on collaterals and throughout the pial artery trees at 8 months of age (**a, b**), the latter which increases directly with diameter. Insets in upper right corners of **a, c, d** show a proximal (2nd order) arteriole at × 50 magnification. By comparison, deposition in 3xTg (**c**) and 1xTg (**d**) mice, which was absent at 8 months of age in both strains, was diffused at 18 months of age in 3xTg and 1xTg mice (**c, d**). Focal plane was set on pial vessels and thus did not image parenchymal Aβ (although both are co-imaged in **c**). Venules and veins had little or no deposition in the three strains at either age. Images are representative of, (for 8 and 18 months of age, respectively): *n* = 6, 5 for the number of 2xTg, 3, 4 for 3xTg, and 3, 3 for 1xTg mice analyzed. Magnification bars, 50 μm

Vascular deposition of Aβ (CAA) on pial/leptomeningeal arteries, arterioles, intracortical PAs, and capillaries is accompanied by inflammation, impaired SMC reactivity, decreased barrier integrity, and apoptosis of vascular wall cells in mouse models of AD; CAA is also evident with both advanced aging and AD in humans and mouse models of AD [6–9, 49–51]. CAA in pial arterial vessels occurs early and progresses with aging in 2xTg mice (present by ~ 6 months [46, 47]), shows early onset in intracortical while minimal-to-absent in pial vessels in 1xTg-AD mice [42, 46], and has not been evaluated in 3xTg mice [46]. Pial collaterals have not been examined in these models. It is possible that the disturbed hemodynamics of low-velocity, bidirectional oscillating flow, and high circumferential wall stress caused by the opposing flow present in pial collaterals at baseline [26, 52], conditions which are well known to be pro-inflammatory in arteries [53, 54], make collaterals especially susceptible to aggregation of Aβ—depending on the genetic mutation(s) and accompanying course of Aβ pathology—in the 2xTg and 3xTg but not 1xTg models, and lead to the collateral rarefaction seen in the former but not 1xTg mice. Our findings do not support this hypothesis. Widespread aggregate CAA (6E10 immunostaining), with its characteristic patchy appearance [49–51, 55, 56], was present throughout the large-caliber branches of the pial artery trees in 8- and 18-month-old 2xTg mice as reported previously [46, 47, 57], and extended distally with less, rather than more, intense deposition on the collaterals (Fig. 3). In contrast, no aggregates of Aβ were detected on pial arteries, arterioles, and collaterals in the 3xTg and 1xTg mice at 8 months of age and were only diffuse and intermittently present at 18 months of age (Fig. 3). Little or no deposition was seen on venules/veins, as reported previously [46, 51], and no vascular Aβ was detected in WT mice. Similar results were obtained with thioflavin-S staining and resorufin. Thus, collaterals do not experience selective early deposition, compared to pial arteries or arterioles, in the 3xTg or 1xTg mice or greater deposition in the 2xTg—a model of AD with extensive early CAA that progresses with age [46, 47, 57]. Likewise, rarefaction in the 3xTg and 2xTg strains (Fig. 1) at 8 months age does not follow the pattern of deposition on pial arteries and collaterals, i.e., present in 2xTg but not 3xTg mice.

### AD-induced collateral rarefaction coincides with the presence of parenchymal total APP/Aβ, CD11b^+^ cells, and HO-1 expression, but does not progress as these parameters increase with aging

**Fig. 4:**
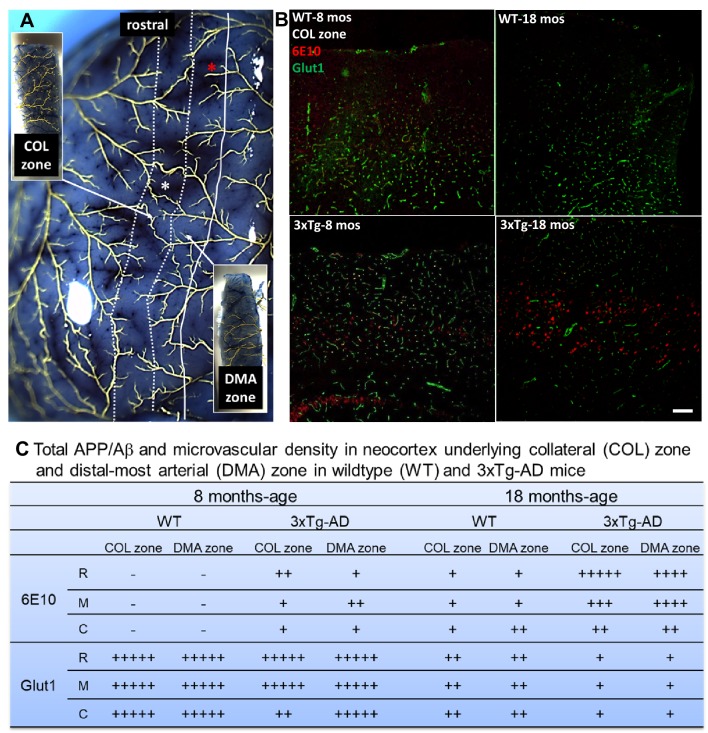
Collateral rarefaction coincides with the presence of intracerebral total APP/Aβ. **a** Pial vasculature maximally dilated, perfused with Evans blue in PBS, and filled with Microfil^R^. White lines delimit the collateral (COL) zone between the MCA (on the left) and ACA trees containing collaterals (white star denotes one) and the distal-most arteriole (DMA) zone of the outer ACA tree containing DMAs (red star denotes one). Insets, examples of tissue blocks of rostral (R), middle (M), and caudal (C) zones that were isolated for slide section histology shown in (**b**), Fig. 5 and Supplemental Fig. IV. **b** Representative sections (10 μm) of middle COL and DMA zones of 8- and 18-month-old wildtype (WT) and 3xTg-AD mice (pial surface is oriented dorsal). **c** Total APP/Aβ score (6E10 immuno-fluorescence) did not differ for cortex underlying the COL zone versus DMA zone in WT or AD mice (*n* ≥ 6 for 8 - and 18-month-old WT and 3xTg mice, respectively) and displayed a positive gradient from caudal to rostral cortex in 3xTg. Total APP/Aβ score burden in AD mice increased between 8 and 18 months of age and also became detectable in 18-month-old WT mice. Score for microvascular density (anti-Glut1, primarily capillaries) was lower in 18 versus 8-month-old WT mice and the decline was greater in AD mice. +−, variably present at a low level (+++++ being a high level) or absent. Magnification bars, 100 μm. Images scored blindly by two independent observers

**Fig. 5:**
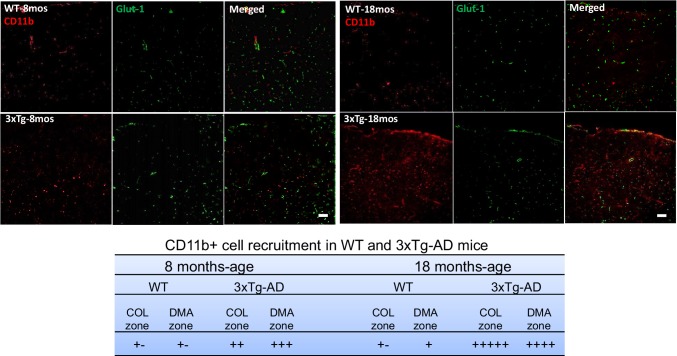
Collateral rarefaction coincides with presence of intracerebral CD11b^+^ cells. Representative sections of neocortex underlying collateral (COL) zone of WT and 3xTg-AD mice (pial surface oriented dorsal). Magnification bars, 50 μm. Table, CD11b^+^ score in AD mice increased between 8 and 18 months of age. DMA, distal-most arteriole; *n* ≥ 6 for 8 -and 18-month-old WT and 3xTg mice, respectively. Images scored blindly by two independent observers

We next examined whether Aβ, CD11b^+^ cell density (a marker for infiltrating monocytes), and activated microglia [5, 9, 58, 59] and expression of the anti-oxidant protein heme oxygenase-1 (HO-1) [60], which are associated with neural and vascular inflammation in AD, are greater in parenchyma underlying the collaterals than in the adjacent cortex. We reasoned this could arise because the collateral/watershed zone between the crowns of the pial artery trees, which includes the underlying cortex whose perfusion is partially supplied by the PAs that branch from the collaterals, is at risk for lower oxygen levels and mitochondrial stress due to its downstream position between the trees (i.e., furthest from the “aorta”) [61]. And that this might lead to greater parenchymal accumulation of Aβ and CD11b^+^ cells [62–64], resulting in rarefaction of collaterals by virtue of their proximity to a more pro-inflammatory environment. Our findings do not support this hypothesis. Cortex underlying the rostral, middle, and caudal collateral zone (“COL zone”) between the MCA and ACA trees, and adjacent cortex underlying the DMAs of the ACA tree (“DMA zone”), was examined in 3xTg and WT mice (Fig. 4). Total APP/Aβ did not differ for COL versus DMA zones in either strain. Controls for 6E10 immunostaining consisted of the positive gradient evident from caudal to rostral cortex in 18-month-old AD mice, reported previously at other ages [40], as well as that Aβ in AD mice increased between 8 and 18 months of age and became detectable in aged WT mice (Fig. 4) as reported by others [6, 8, 37, 40]. Also in agreement with previous studies [49, 65, 66], microvascular density (primarily capillaries), indicated by the endothelial cell (EC) marker Glut1, was lower in 18 versus 8-month-old WT mice, and this age-associated decline was greater in AD mice (Fig. 4). Microvascular density did not differ between collateral and DMA zones for WT and AD mice at 8 months of age (except for the caudal collateral zone of AD mice). Findings similar to the above for Aβ were obtained for CD11b^+^ cell density (Fig. 5) and expression of HO-1 (Supplemental Fig. IV). CD11b^+^ cells and HO-1, which were low or undetectable in WT mice, were present at comparable levels in the collateral and DMA zones of 3x-Tg mice at 8 months of age and increased further by 18 months of age.

Increased levels of total APP/Aβ, CD11b^+^ cells and HO-1 present at 18 months of age were not accompanied by additional rarefaction (Fig. 1), even though additional rarefaction occurs in 31-month-old B6 mice (i.e., 22% and 30% decrease in number and diameter) [29]. This suggests that AD-induced rarefaction reaches a maximum by 8 months of age. However, mice older than 18 months of age would have to be studied to confirm this. The absence of effect of AD on microvascular density in cortex at 8 months of age (Fig. 4), along with absence of effect on number or diameter of DMAs and PAs, further underscores that AD causes selective rarefaction of collaterals at an early age.

### AD-induced rarefaction is associated with increased markers of inflammation, oxidative stress, and aging of collateral wall cells

**Fig. 6:**
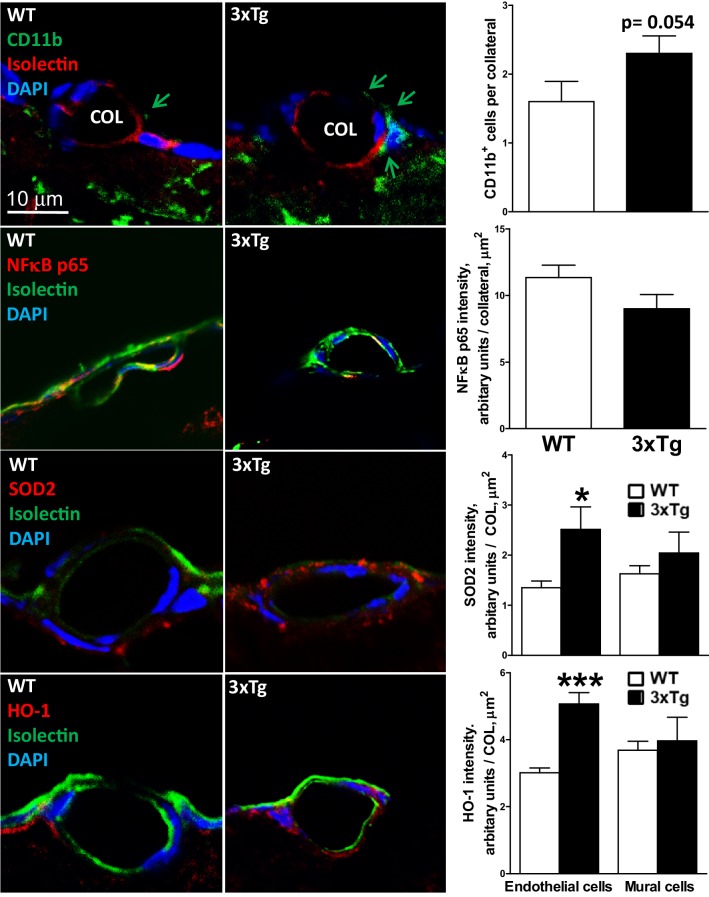
AD-induced collateral rarefaction is associated with increased markers of inflammation and oxidative stress. Collaterals (COL) were identified by lumen filled with Microfil and roadmap of tissue blocked for histology per Fig. 4a. Isolectin identifies endothelial cells, DAPI identifies nuclei. 8-month-old WT and 3xTg mice. Increased CD11b^+^ perivascular cells (arrows), a marker of inflammation, and increased expression of the anti-oxidant proteins SOD2 and heme oxygenase-1 (HO-1), markers for the presence of increased oxidative cell stress. *n* = 5 mice for each bar. *^,^**^,^***p < 0.05, 0.01, 0.001 versus WT. Magnification bar (first panel) same for all panels. Images scored blindly by two independent observers

**Fig. 7:**
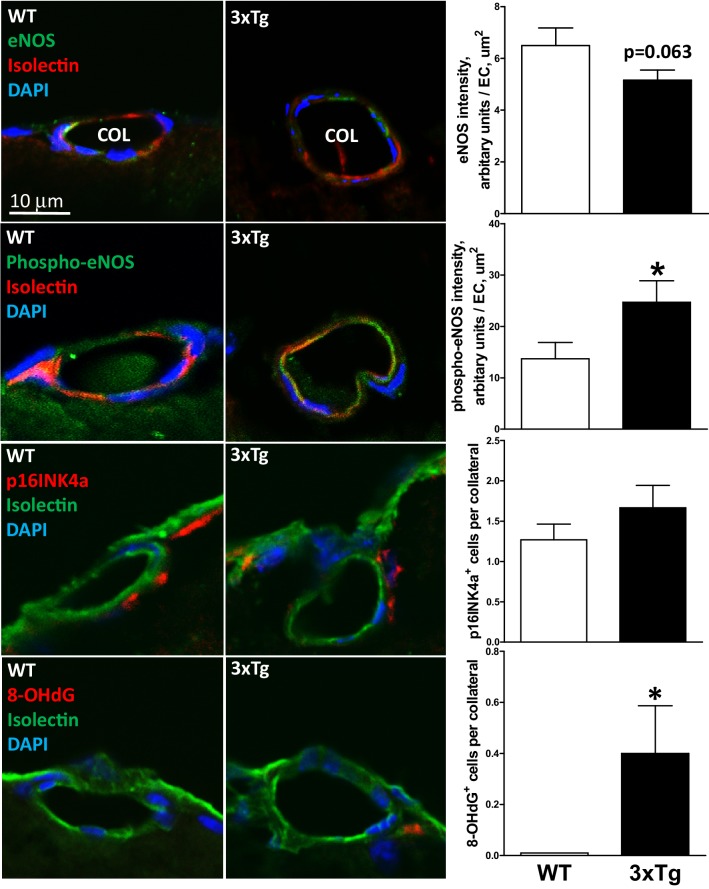
AD-induced rarefaction is associated with changes in eNOS and increased markers of aging of collateral wall cells. Collaterals (COL) were identified by lumen filled with Microfil and roadmap of tissue blocked for histology per Fig. 4a. Isolectin identifies endothelial cells, DAPI identifies nuclei. 8-month-old WT and 3xTg mice. Increased 8-OHdG is a marker for cellular aging. *n* = 5 mice for each bar. *p < 0.05 versus WT. Magnification bar (first panel) same for all panels. Images scored blindly by two independent observers

Accumulation of intra- and extracellular Aβ, which occur with advanced aging and are accelerated in AD, promote oxidative stress and inflammation in the parenchyma and cerebral vasculature [2–9, 58–63]. It is possible that these conditions combine with the above-mentioned unique local hemodynamic environment that collateral wall cells reside in: The low/disturbed shear stress, high wall stress, and reduced PO_2_ (that is likely close to parenchymal PO_2_ that is ~ 14 mmHg at rest [67]) experienced by collaterals favor vascular inflammation and endothelial dysfunction when present elsewhere in the circulation [53, 54]. This environment may underlie the increased cell proliferation/turnover of collateral ECs and length/tortuosity of collaterals compared to DMAs—the latter a hallmark of collaterals—that increase with aging in B6 mice and coincide with collateral rarefaction [29, 31, 32]. The inflammation and oxidative stress caused by the AD pathology present in the 3xTg and 2xTg mice could increase the already-accelerated proliferation and aging of collateral wall cells present in healthy mice, leading to the greater tortuosity (Fig. 2) and earlier onset of rarefaction (Fig. 1). The findings in Figs. 6 and 7 support this hypothesis. Collaterals of 8-month-old 3xTg mice had increased perivascular CD11b^+^ cells and expression of SOD2 and HO-1. NF_K_B p65 was not altered. Total eNOS trended lower (*p* = 0.063), consistent with the above changes indicative of an increase in inflammatory and oxidative stress, and phospho-eNOS increased possibly as a compensation for the lower total eNOS. Markers of cellular aging/proliferative senescence, 8-OHdG and p16^INK4a^, increased or trended as such, consistent with the increase in collateral tortuosity in the AD mice (Fig. 2).

### Rarefaction is inhibited in AD mice deficient in CX_3_CR1 or overexpressing eNOS

**Fig. 8:**
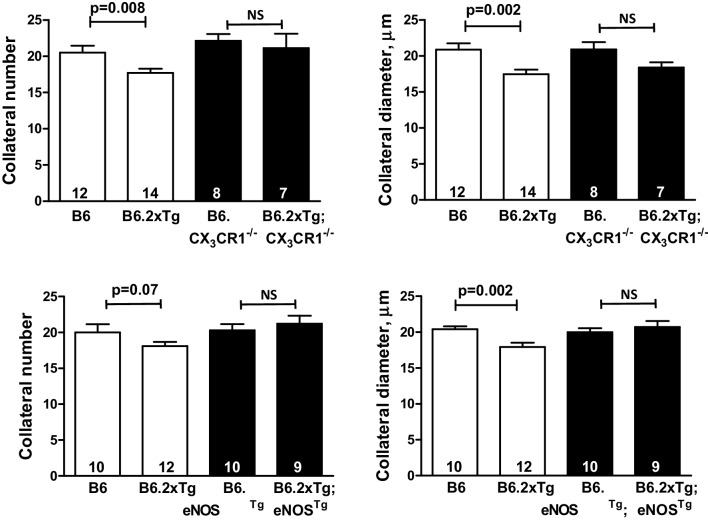
AD-induced rarefaction is lessened in AD mice deficient in CX_3_CR1 and prevented by increased expression of eNOS. CX_3_CR1^−/−^, receptor knockout mice. eNOS^Tg^, endothelial nitric oxide synthase transgenic mice. These strains were crossed with B6.2xTg-AD mice. White bars (data from Fig. 1 to aid interpretation) and black bars indicate separate cohorts of mice. Top panels, 8 months old; bottom panels, 18 months old. *NS* non-significant

AD-induced amyloidosis causes release of fractalkine (CX_3_CL1) from neurons, which by stimulating its receptor CX_3_CR1 that is present on microglia and several leukocyte types, promotes neural and vascular inflammation and reduced eNOS and nitric oxide (NO) [4, 5, 68–70]. Moreover, reduced eNOS/NO also occurs with aging and other vascular risk factors and has been causally linked to the collateral rarefaction that accompanies them [29–32, 70]. To test for involvement of fractalkine and eNOS deficiency in AD-induced collateral rarefaction, we crossed B6.2xTg mice (since 3xTg mice are on a mixed B6-129Sv background) with B6.CX_3_CR1^−/−^ mice and also with B6.eNOS transgenic mice (the latter have a twofold increase in NO production [32]). Since various treatments are available that maintain or increase endothelial NO bio-availability, B6.2xTg; B6.eNOS transgenic mice were examined at 18 months age (~ 56 human-year equivalents [44]) to better simulate the age when familial AD becomes apparent in humans. Loss of collateral number was prevented in 2xTg; CX_3_CR1^−/−^ mice, and loss of both number and diameter were prevented in 2xTg; eNOS^Tg^ mice (Fig. 8). These findings demonstrate that collateral rarefaction in young-adult AD mice involves fractalkine signaling and suggest this leads to inflammation-induced decline in eNOS/NO.

## Discussion

This study yielded several important findings. Collateral rarefaction occurred by similar amounts in 2xTg and 3xTg mice when examined at 8 months of age (~ 28 human-year equivalents [44]). No significant rarefaction was observed at 1 month of age, nor had the rarefaction at 8 months progressed significantly further at 18 months in the cohorts examined. Thus, rarefaction of collaterals in AD occurred well before that seen with natural aging, where declines in collateral diameter and number became evident at 16 and 24 months of age (~ 52 and 72 hye), respectively [29, 32]. Rarefaction was accompanied by an increase in collateral tortuosity (31%), which reflects an increase in EC proliferation [29, 32], and an increase in relative resistance of the collateral network (2.9-fold) and infarct volume after pMCAO (91%). Similar changes also occurred with aging [36, 40]. No significant rarefaction was seen in 8-month-old 1xTg mice, and only a small decrease in collateral number was present at 18 months of age. This difference in the models may reflect different mechanisms of AD pathology induced by the *APP*SwDI transgene present in the 1xTg model, which leads to Aβ aggregation on intracerebral capillaries and PAs [42, 46]. Rarefaction occurred irrespective of Aβ aggregation on collaterals or pial arteries, i.e., extensive deposits were present at 8 months of age in the 2xTg but absent in the 3xTg (and 1xTg) mice. However, rarefaction was accompanied by elevated levels in collateral wall cells of proteins associated with increased oxidative stress, inflammation, and aging. These conditions are known to impair eNOS-derived NO [6, 29, 31, 32, 36, 37, 53, 54, 70], which is an important collateral “maintenance” factor that protects against age-associated rarefaction [29–32]. These same conditions also accompany collateral rarefaction that occurs with aging or vascular risk factors such as hypertension and metabolic syndrome [29–32]. Consistent with the above findings, rarefaction was lessened in 2xTg mice deficient in CX_3_CR1 and prevented by increased expression of eNOS even when examined at 18 months of age. Interestingly, increased eNOS achieved by chronic exercise training also prevented rarefaction and the increase in infarct volume after pMCAO that occur with advanced aging [32].

Although there is no published work on collaterals for comparison to our findings, Dorr and co-workers [49] examined PAs in TgCRND8 AD mice at 3, 6, and 12 months of age and found no significant change in length or diameter in AD or WT mice (number was not reported). A small increase in tortuosity and reduced vasodilation to CO_2_ was seen in 12-month-old AD mice. Increased pial vessel tortuosity, decreased intracerebral vessel density, degeneration of SMCs, and decreased blood–brain barrier integrity have been reported in humans with AD [2–7, 37, 49, 50, 56, 64–66].

Collateral rarefaction is likely an important contributor to the greater infarct volumes that we observed in AD mice, since collateral conductance and lesion size following pMCAO are primarily dependent on collateral number and diameter [23, 24]. Previous studies have reported that larger lesions and worse outcomes occur following acute ischemic stroke; however, the responsible mechanisms were unclear, thus prompting this study. In humans with AD, combined presence of *ApoE-ε4* allele and CAA on autopsy increased the odds of having cortical infarction 3.2-fold [11]. Since atherosclerosis or small vessel disease did not account for the association, the authors concluded that “the mechanism underlying the association remains enigmatic.” Other studies have found that AD increases the risk of ischemic stroke [14], although this was not confirmed elsewhere [12, 13]. In 3–4-month-old APP695Swe transgenic (Tg2576) mice, which had elevated Aβ but not perivascular Aβ deposits or neuritic plaque, blood flow in the penumbra following pMCAO was reduced twofold and infarct volume measured 24 h later was increased 32% [15]. The authors speculated that the findings might result from impaired collateral blood flow. Milner et al. [16] reported that young Tg2576 mice had increased infarctions after transient MCAO, and that 15-month-old Tg2576 with established CAA and neuritic plaques also had larger infarct volume and greater deficit in blood flow immediately after occlusion. The authors suggested that the latter results “likely relate to the inability of nearby CAA-laden cerebral arterioles to provide collateral blood flow through autoregulatory vasodilation.” Infarct volume after pMCAO was 36% larger in 9-month-old APP/PS1dE9 AD mice which had extensive Aβ deposits and microgliosis [17]. Similarly, in 8- and 20-month-old APP751 transgenic mice which have elevated Aβ but no significant CAA or neuritic plaque, infarct volumes were 34% and 41% larger than controls [18]. Iadecola and co-workers [19] reported that 3–4-month-old APP_695_Swe mice (FVB/*n* background) with high levels of Aβ had a 42% greater decrease in blood flow in the outer MCA tree and 32% larger infarct volume 24 h after pMCAO than WT controls. Other investigators found that 18-month-old mice transgenic for mutant *APP*, but not for *PSEN-1*, sustained larger infarct volumes after photothrombotic occlusion [20]. A lack of effect on infarct volume in *PSEN-1* transgenic mice was also reported by others [18, 71, 72]. Our finding that a similar amount of collateral rarefaction occurred at 8 months of age in models of AD with (2xTg) and without (3xTg) CAA is congruent with the above studies and suggests that rarefaction of pial collaterals occurs early in the course of the disease in association with diffuse parenchymal amyloidosis, predisposing to increased infarctions.

Microvascular density within the parenchyma, representing primarily capillaries, was reduced in 8- and 18-month-old 3xTg mice. This could also contribute to the increased infarction that we observed after MCAO. However, based on previous studies that used genetic methods to alter collaterogenesis and thus increase or decrease collateral number and diameter without altering non-collateral vessels [24], the reductions in collateral number and diameter that occurred in the 8-month-old 3xTg mice would, alone, increase infarct volume by ~ 100%. This is close to—and not less than—the 91% increase that we observed, indicating that the increased infarct volume in the AD mice can be primarily attributed to collateral rarefaction. However, lower capillary density at baseline and/or inhibition of ischemic angiogenesis in the peri-infarct region could contribute to poorer functional outcomes in AD after acute ischemic stroke. Decreased capillary density has been reported in humans with AD [65, 66], in double transgenic AD mice [49, 73], and in ArcAbeta AD mice with significant CAA [74]. APP Aβ binds VEGF and Flk1 (VEGFR2) [75], and inhibits angiogenesis in vitro and in vivo [76, 77]. At 18 months of age, microvascular density was reduced in WT mice (Fig. 4), as is known to occur with advanced aging [49, 65, 66]. Reduced capillary density in AD and aging is congruent with loss of synapses, neurons, and brain mass that accompany both conditions.

What might underlie the selective rarefaction of collaterals seen early in the progression of AD and its prevention by increased expression of eNOS? Collateral rarefaction that occurs with aging and other vascular risk factors [29–31], which are accompanied by oxidative stress and endothelial/eNOS dysfunction, is inhibited—at least in the case of aging—by daily aerobic exercise, which increases eNOS expression [40]. Furthermore, robust eNOS activity is required for maintenance of collateral number during adulthood [30, 31]. Thus, the finding that Aβ, itself, causes oxidative stress, inflammation, and endothelial/eNOS dysfunction [4–7, 9, 15, 16, 37], provides one mechanism that may underlie AD-induced collateral rarefaction. Recent findings provide additional clues. Genetic deficiency of eNOS, in both in vitro studies of human brain microvascular ECs and in vivo studies in mice, causes increased endothelial expression of APP and β-secretase-1 (BACE-1), appearance of vascular and parenchymal Aβ, and cognitive deficits that are prevented by administration of l-arginine or a PDE5 inhibitor [70]. The latter also improves cognitive function in APP-PS1 and Tg2576 AD mice without affecting brain amyloid burden, suggesting a vascular contribution p [70]. In cerebral vessels from Tg2576 AD mice, increased expression of Aβ is accompanied by increased NADPH oxidase and superoxide anion and uncoupled eNOS [70, 78]. Moreover, physiologic expression of APP in brain ECs is required to maintain normal eNOS expression, leading to the suggestion that disturbed metabolism of APP in mutant APP models of AD may lead to impaired expression of eNOS and vascular dysfunction [79]. Furthermore, senescence of human brain ECs was accompanied by increased levels of BACE-1 and Aβ_1−40_, and the latter was reversed by treatment with a BACE-1 inhibitor [80]. And absence of eNOS increased Aβ pathology in Tg-5xFAD mice [81]. Deficient VEGF signaling may also contribute to collateral rarefaction in AD. VEGF contributes to collateral formation during development and stabilization and maturation during growth to adulthood [25, 27]. Aβ binds VEGF and Flk1 [75] and inhibits Flk1’s downstream signaling pathway that includes eNOS, which is required for EC survival [75, 76], and accelerates EC senescence [77]. Inhibition of Flk1 and eNOS signaling and increased EC senescence are associated with increased production of reactive oxygen species and apoptosis of ECs [82–84].

Rarefaction was lessened in 2xTg mice deficient in CX_3_CR1, the receptor for fractalkine/CX_3_CL1. CX_3_CR1 is present on microglia, patrolling monocytes, NK cells, and cytotoxic T cells, and its signaling has been implicated in AD [2, 5, 7, 9, 58, 59, 68, 69, 78]. Expression of fractalkine is induced primarily in neurons but also astrocytes, ECs, and SMCs activated by intraneuronal and extracellular Aβ and other inflammatory factors. Binding of Aβ to microglial cells induces neuroinflammation and micro- and astrogliosis involving soluble fractalkine-mediated recruitment of activated CD11b^+^ microglia and peripheral immune cells (the latter’s CX_3_CR1 ligate membrane-bound fractalkine on activated ECs and promote transmigration) that release IL6/1β, TNFα, free radicals, and other cyto/chemokines [2, 5, 7, 9, 58, 59, 68, 69, 78]. Of note, fractalkine augmented production of reactive oxygen species in ECs and SMCs, resulting in impaired eNOS-NO that was reversed by anti-oxidant treatment [68]. Furthermore, deficiency in CX_3_CR1 increased microglial activation and Aβ clearance in 2xTg mice, lessened neuronal loss in 3xTg mice [69], and reduced Aβ levels in two mouse models of AD in an allele-dose dependent manner [85]. The above findings support a role for fractalkine in AD pathology and suggest that the decrease in collateral rarefaction that we observed in 2xTg; CX_3_CR1^−/−^ mice could result from reduced Aβ and/or tempered inflammatory signaling driven by intracerebral and/or vascular Aβ. However, depending on context, fractalkine may also exert a protective role [5]. For example, fractalkine is reduced in hippocampus and cortex of 9- and 17-month-old Tg2576 mice [86] and levels of both ligand and receptor decrease with aging in association with a decline in the ability of microglia to mount a normal response to injury [87].

Like the potential involvement of eNOS and VEGF in AD pathology and maintenance of collaterals in the results that we obtained in eNOS^Tg^; CX_3_CR1^−/−^ mice, interactions between fractalkine and VEGF may also be involved in the inhibition of rarefaction in the CX_3_CR1^−/−^; 2xTg mice. Fractalkine increases ADAMTS-1 (a disintegrin and metalloprotease domain with TSP repeats protein-1), which binds and sequesters VEGF, and also cleaves matrix-bound thrombospondin-1—itself increased by fractalkine—to release its anti-angiogenic and apoptosis-sensitizing EC activities [88, 89]. Both ADAMTS-1 and thrombospondin-1 are highly expressed in ECs and SMCs. Reduction in fractalkine signaling may thus oppose collateral rarefaction induced by Aβ-mediated inflammation of collateral wall cells. Additional mechanisms may also contribute to AD-induced rarefaction. Impaired autoregulation of blood flow in AD [10, 49] could increase the magnitude of disturbed shear stress and the already-increased wall stress that are unique to the collateral hemodynamic environment [26, 52], leading to endothelial dysfunction, enhanced proliferative EC senescence, and collateral rarefaction. In addition, oxygen level adjacent to collateral wall cells is predictably low due to their disturbed hemodynamics, compared to other pial arterial vessels, which may favor increased production of Aβ by their ECs and SMCs [61–65] that could promote rarefaction according to the following: Since flow/shear stress in collaterals at baseline ebbs back and forth with a net value of zero [26, 52], and given that collaterals are the furthest pial arterial outposts from the aorta [61], the PO_2_ in collateral blood is predictably lower than elsewhere in the pial arterial vasculature. And hypoxia has been found in vitro, in vivo in animal models, and ex vivo in human brain to increase Aβ production and decrease degradation and clearance [63].

Based on our results and the above findings of others it is possible that, early in the course of AD, diffuse neuronally derived Aβ (and possibly Aβ released from ECs [70]), which is known to cause oxidative stress and release of inflammatory mediators including fractalkine from neurons, astrocytes, and activated ECs and SMCs [2, 5, 7, 9, 58, 59, 68, 69, 78] leads to peri-collateral recruitment of CD11b^+^ immune cells, lower eNOS-NO and VEGF signaling and other changes in collateral ECs and SMCs associated with vascular inflammation (Fig. 7). Given that eNOS-NO is well known to oppose vascular inflammation, adhesion of platelets and leukocytes, and proliferation and aging of ECs [53, 54], we hypothesize that the above conditions have an especially detrimental effect on collateral ECs—similar to the elevated proliferation of collateral ECs, increased tortuosity, and eventual collateral rarefaction that occurs with aging and chronic risk factor presence [29–31] which, themselves, cause increased vascular oxidative stress, inflammation, and endothelial dysfunction [6, 53, 54, 85]. As well, Aβ has been shown to directly increase EC proliferation in vitro [97]. Collateral pruning could thus extend from chronic, low-grade, Aβ inflammation-induced accelerated EC proliferative senescence in association with decreased eNOS, VEGFA-Flk1 signaling [75], and changes in other downstream effector proteins, resulting in EC destabilization, pericyte loss, increased perivascular leukocytes, EC and SMC apoptosis, decline in vessel diameter, and eventual vessel end-closure, and loss of the collateral anastomosis. Interestingly, if basement membrane sleeves/strings persist after collateral pruning, as has been reported with other types of vessel pruning [65, 66], it might be possible to induce collaterals that have been lost to re-grow through such extant scaffolds.

Like all scientific studies, our results are conditional and subject to limitations. The mice studied herein are highly aggressive models of human familial AD and do not model the late-onset sporadic form of the disease that is much more common in humans. A second limitation is that our studies are in mice, not humans. A third limitation is that the interpretation of the data shown in Fig. 8 for the 2xTg; CX_3_CR1^−/−^ and 2xTg; eNOS transgenic mice would be more compelling, regarding mechanism, if these genetic crosses had also been evaluated for intracerebral Aβ, CD11b^+^ cells and the measures shown in Figs. 6 and 7 for inflammatory, oxidative stress, and aging markers. Not only is this an important control measurement, but how Aβ dose relates to collateral dysfunction might provide insight into how regional Aβ changes contribute to worsened blood flow and ischemia outcomes. A fourth limitation is that the rescue outcomes observed in the B6.2xTg; B6.CX_3_CR1^−/−^ and B6.2xTg; B6.eNOS^Tg^ mice were not tested for confirmation in the 3xTg model. This was because the latter are on a mixed B6-129Sv background, thus transferring the knockout and transgenic alleles onto a near-pure B6-129Sv background would require 10 generations of backcrossing. A fifth limitation is that comparison of parenchymal Aβ between the three AD mouse models at 8 and 18 months of age would have aided interpretation (i.e., Do 1xTg express less Aβ than 2xTg and 3xTg mice?). Unfortunately, we are unaware of a previous publication that compared 6E10 staining in the three mouse models examined herein. However, at 8 months of age, collateral rarefaction had occurred to similar degrees in 8-month-old 2xTg and 3xTg mice, but was absent in 1xTg mice (Fig. 1). We therefore examined whether the rarefaction was associated with parenchymal Aβ in 3xTg mice (Fig. 4). A-beta was present at 8 months of age but was much stronger by 18 months of age, yet rarefaction was not greater at 18 months versus 8 months of age. As stated in Results and in Discussion, this indicates that rarefaction occurs early in 3xTg mice when Aβ accumulation is moderate and does not become more severe when Aβ is much more prevalent, suggesting that the relationship is not a simple 1-to-1. An addition caveat is that other changes resulting from transgenic co-expression of mutant APP and presenilin-1, in addition to collateral rarefaction, could contribute to the increased infarct volume that we observed. These include a possible increase in neuronal sensitivity to ischemia or increase in resistance within the MCA territory to collateral-dependent perfusion after pMCAO that could arise from impaired vasodilation, leukocyte and platelet adhesion, rheological changes, and edema. However, data obtained with different targeted mutations of the gene, *Rabep2*, that cause graded reductions in collateral formation during development and thus fewer collaterals of smaller diameter and increased infarct volumes in the adult [24], do not support this caveat. Those data show that the magnitude of reduction in the number and diameter of collaterals that occurred in the 8-month-old 3xTg mice in the present study would, alone, increase infarct volume by ~ 100%, which is close to the 91% increase that we observed.

In conclusion, we found that two mouse models of AD caused selective loss of collateral number and diameter between 1 and 8 months of age and increased lesion volume following pMCAO. These findings add collateral rarefaction to the other vascular impairments that accompany and may contribute to the progression of AD [6–10, 56, 70, 76–78, 82–84]. They also identify a mechanism that may contribute to the increased stroke risk, lesion size, and worse outcomes following acute ischemic stroke in AD patients and mouse models of AD. It will be important in future studies to examine collateral rarefaction in a next-generation mouse model(s) of sporadic AD [90, 91], and if confirmed, test for association of collateral score in acute ischemic stroke patients with and without AD. Additional studies are required to identify the cell types that express fractalkine/CX_3_CL1 and its CX_3_CR1 receptor that are involved in the rarefaction, and to identify the pathway activated by CX_3_CR1, as well as other mechanisms, that lead to the impaired eNOS/NO known to occur in AD [6, 36–38, 70, 83] and that when countered in our 2xTg; eNOS transgenic mice prevented rarefaction. Many inflammatory mediators released by activated microglia and peripheral immune cells impair eNOS/NO and accelerate proliferation and apoptosis of ECs and SMCs. It would also be interesting to examine whether strategies to chronically augment eNOS/NO (e.g., PDE5 inhibitor, NO donor, l-arginine, regular aerobic exercise [32, 92]), if begun early in AD, inhibit collateral rarefaction. Previous work in mice has shown that maintenance of collaterals depends on robust expression of eNOS at baseline [30, 31], and that aging and other vascular risk factors that cause endothelial dysfunction lead to collateral rarefaction in brain and other tissues [29–32]. The current study adds AD to this list of conditions and diseases that may adversely affect the cerebral collateral circulation.

## Electronic supplementary material

Below is the link to the electronic supplementary material.

Supplementary material 1 (PPTX 6391 KB)

Supplementary material 2 (DOCX 38 KB)

## Abbreviations

Aβ: Amyloid precursor protein-β peptides (amyloid-β)
ACA: Anterior cerebral artery
AD: Alzheimer’s disease
APP: Amyloid precursor protein
B6: C57BL/6J inbred mouse strain
CAA: Cerebral amyloid angiopathy
COL: Collateral
CX_3_CL1: The ligand, also known as fractalkine, that binds CX_3_CR1
DMA: Distal-most arteriole in a cerebral artery tree
EC: Endothelial cell
HO-1: Heme oxygenase-1
hye: Human-year equivalents (of age)
MCA: Middle cerebral artery
eNOS: Endothelial nitric oxide synthase
NO: Nitric oxide
PA: Penetrating arteriole (perforator)
PCA: Posterior cerebral artery
pMCAO: Permanent distal (post-lenticulostriate) M1-MCA occlusion
SMC: Smooth muscle cell
SOD2: Superoxide dismutase-2
WT: Wildtype control littermates
1xTg-AD: Single transgenic AD mouse strain, *APP*SwDI
2xTg-AD: Double transgenic AD mouse strain, *APP*695, *PSEN-1*
3xTg-AD: Triple transgenic AD mouse strain, *APP*Sw, *PSEN-1, MAPT* (Tau)
8-OHdG: 8-hydroxy-2′-deoxyguanosine
