# Supplementary material for: Mouse models of Alzheimer’s disease cause rarefaction of pial collaterals and increased severity of ischemic stroke
Source: Angiogenesis. 2018 Dec 5;22(2):263–79. doi: 10.1007/s10456-018-9655-0 (PMC6475514; doi:10.1007/s10456-018-9655-0)
Supplement: Supplementary file 1 — Supplementary material 1 (PPTX 6391 KB) [file 10456_2018_9655_MOESM1_ESM.pptx]

## Slide 1
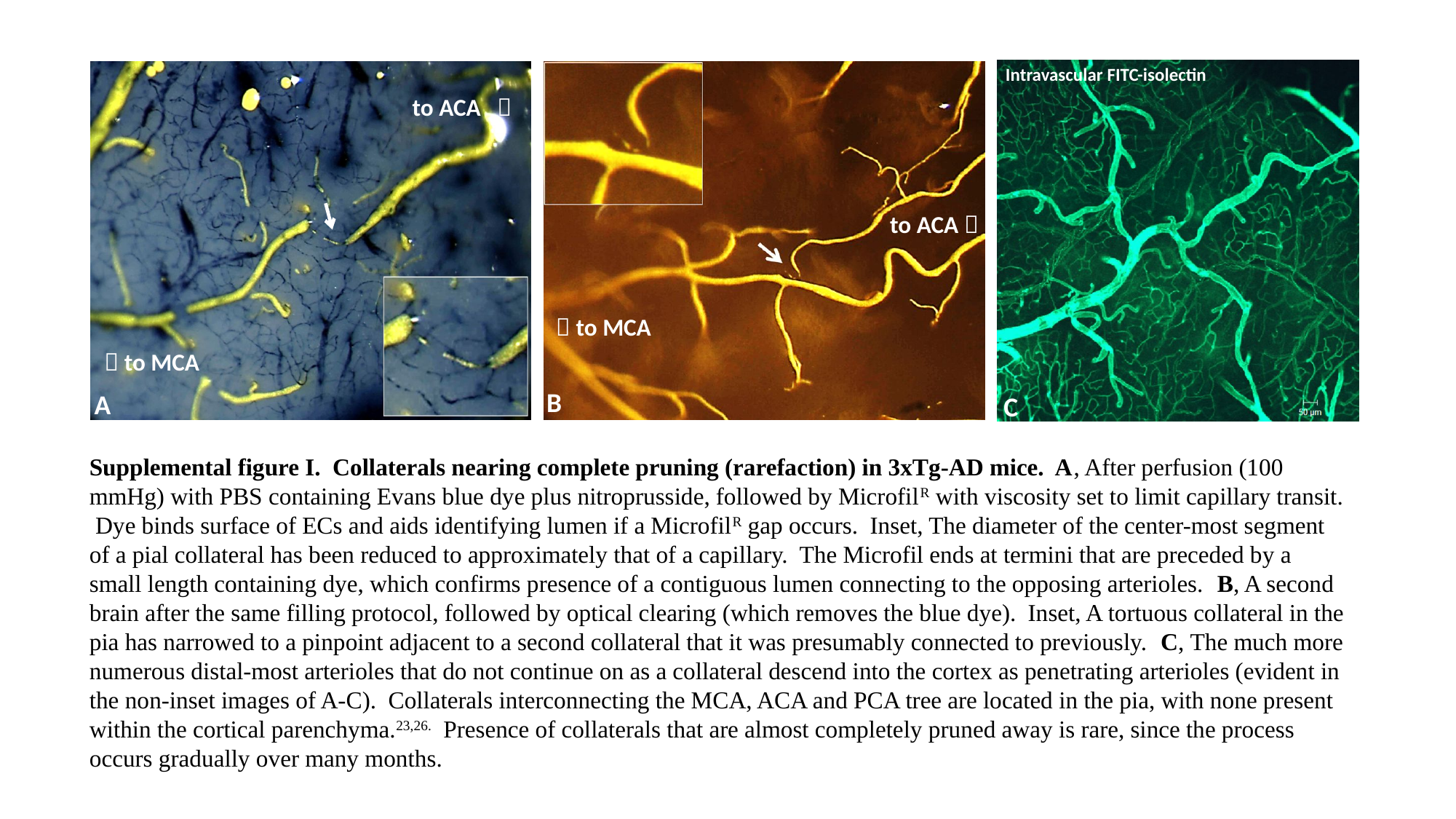

Intravascular FITC-isolectin
to ACA 
to ACA 
 to MCA
 to MCA
B
A
C
Supplemental figure I. Collaterals nearing complete pruning (rarefaction) in 3xTg-AD mice. A, After perfusion (100 mmHg) with PBS containing Evans blue dye plus nitroprusside, followed by MicrofilR with viscosity set to limit capillary transit. Dye binds surface of ECs and aids identifying lumen if a MicrofilR gap occurs. Inset, The diameter of the center-most segment of a pial collateral has been reduced to approximately that of a capillary. The Microfil ends at termini that are preceded by a small length containing dye, which confirms presence of a contiguous lumen connecting to the opposing arterioles. B, A second brain after the same filling protocol, followed by optical clearing (which removes the blue dye). Inset, A tortuous collateral in the pia has narrowed to a pinpoint adjacent to a second collateral that it was presumably connected to previously. C, The much more numerous distal-most arterioles that do not continue on as a collateral descend into the cortex as penetrating arterioles (evident in the non-inset images of A-C). Collaterals interconnecting the MCA, ACA and PCA tree are located in the pia, with none present within the cortical parenchyma.23,26. Presence of collaterals that are almost completely pruned away is rare, since the process occurs gradually over many months.

## Slide 2
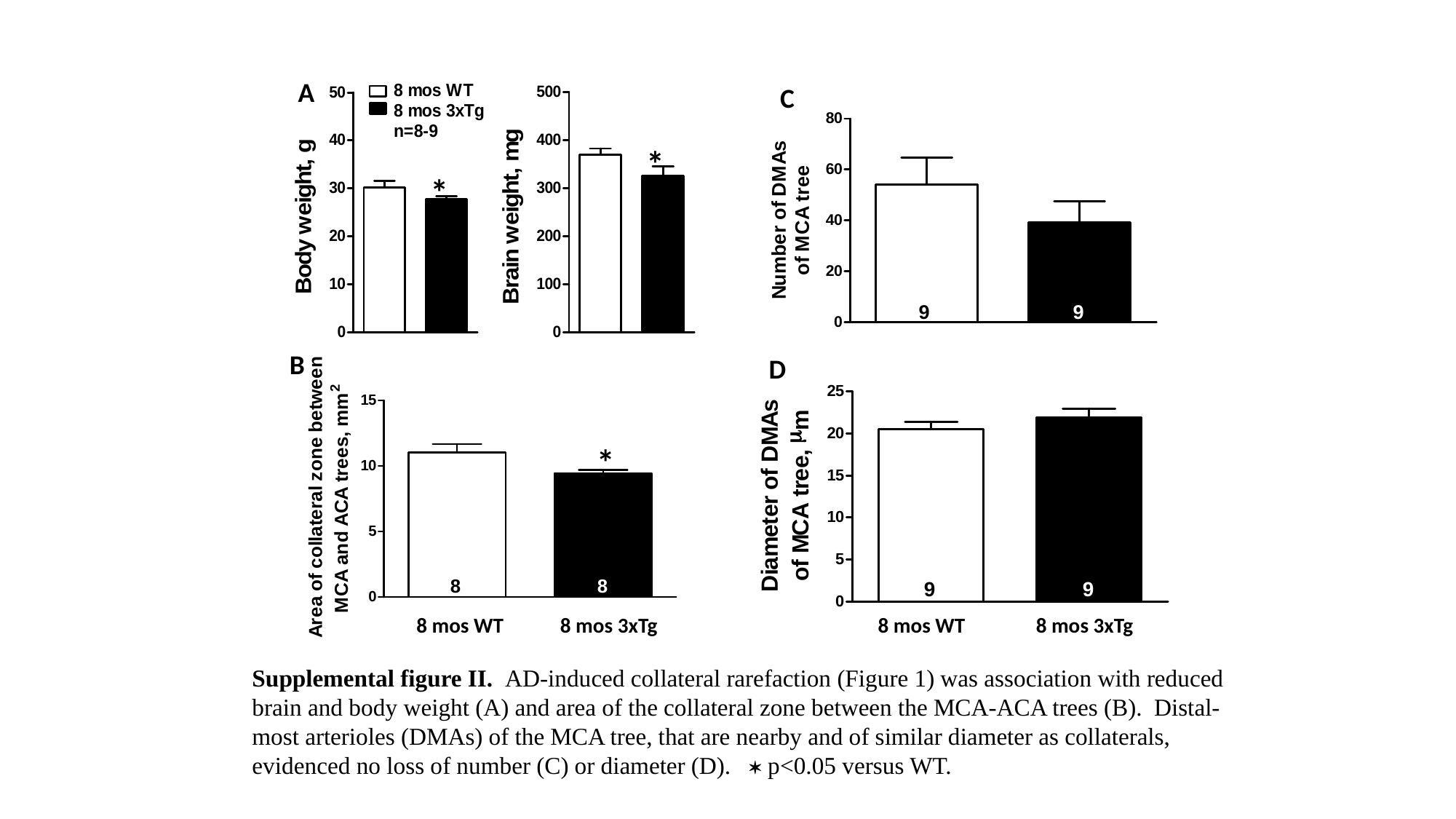

*
*
A
C
*
D
 8 mos WT 8 mos 3xTg
B
*
 8 mos WT 8 mos 3xTg
Supplemental figure II. AD-induced collateral rarefaction (Figure 1) was association with reduced brain and body weight (A) and area of the collateral zone between the MCA-ACA trees (B). Distal-most arterioles (DMAs) of the MCA tree, that are nearby and of similar diameter as collaterals, evidenced no loss of number (C) or diameter (D).  p<0.05 versus WT.

## Slide 3
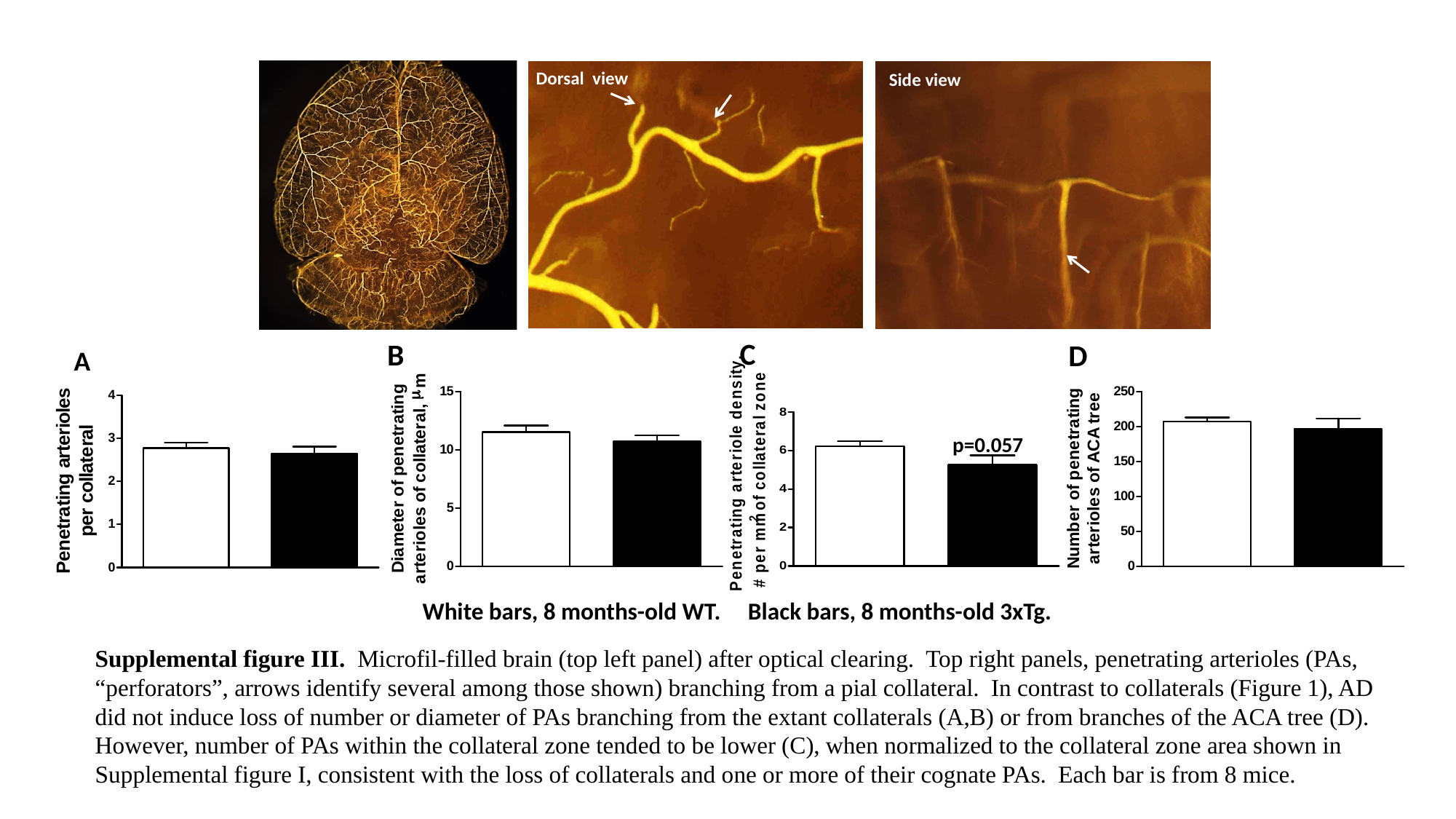

Dorsal view
Side view
C
p=0.057
B
D
A
 White bars, 8 months-old WT. Black bars, 8 months-old 3xTg.
Supplemental figure III. Microfil-filled brain (top left panel) after optical clearing. Top right panels, penetrating arterioles (PAs, “perforators”, arrows identify several among those shown) branching from a pial collateral. In contrast to collaterals (Figure 1), AD did not induce loss of number or diameter of PAs branching from the extant collaterals (A,B) or from branches of the ACA tree (D). However, number of PAs within the collateral zone tended to be lower (C), when normalized to the collateral zone area shown in Supplemental figure I, consistent with the loss of collaterals and one or more of their cognate PAs. Each bar is from 8 mice.

## Slide 4
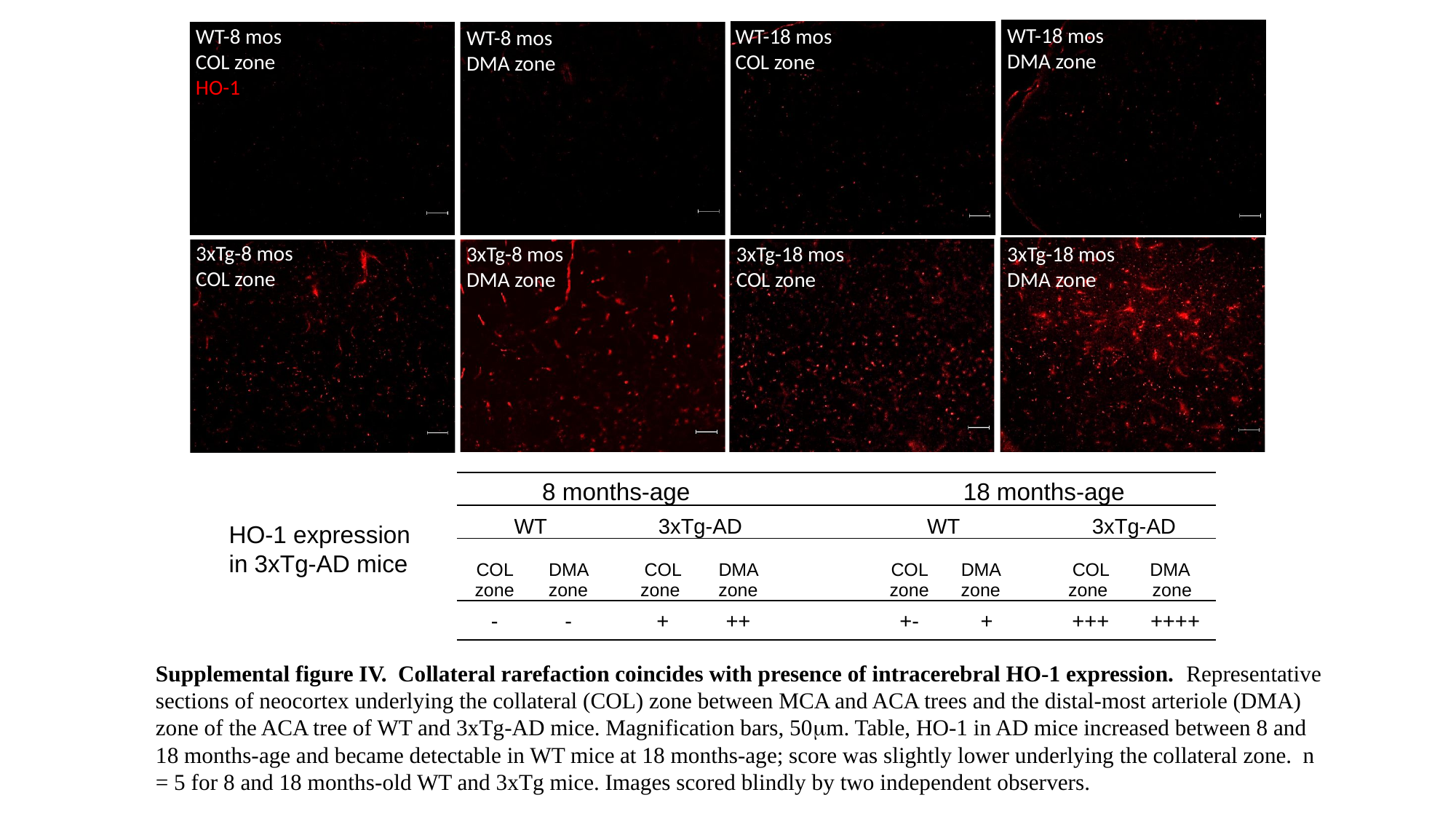

WT-18 mos
DMA zone
WT-18 mos
COL zone
WT-8 mos
COL zone
HO-1
WT-8 mos
DMA zone
3xTg-18 mos
COL zone
3xTg-18 mos
DMA zone
3xTg-8 mos
DMA zone
3xTg-8 mos
COL zone
| 8 months-age | | | | | | 18 months-age | | | | |
| --- | --- | --- | --- | --- | --- | --- | --- | --- | --- | --- |
| WT | | | 3xTg-AD | | | WT | | | 3xTg-AD | |
| COL zone | DMA zone | | COL zone | DMA zone | | COL zone | DMA zone | | COL zone | DMA zone |
| - | - | | + | ++ | | +- | + | | +++ | ++++ |
HO-1 expression in 3xTg-AD mice
Supplemental figure IV. Collateral rarefaction coincides with presence of intracerebral HO-1 expression. Representative sections of neocortex underlying the collateral (COL) zone between MCA and ACA trees and the distal-most arteriole (DMA) zone of the ACA tree of WT and 3xTg-AD mice. Magnification bars, 50μm. Table, HO-1 in AD mice increased between 8 and 18 months-age and became detectable in WT mice at 18 months-age; score was slightly lower underlying the collateral zone. n = 5 for 8 and 18 months-old WT and 3xTg mice. Images scored blindly by two independent observers.
