# Supplementary material for: Mouse models of Alzheimer’s disease cause rarefaction of pial collaterals and increased severity of ischemic stroke
Source: Angiogenesis. 2018 Dec 5;22(2):263–79. doi: 10.1007/s10456-018-9655-0 (PMC6475514; doi:10.1007/s10456-018-9655-0)
Supplement: Supplementary file 2 — Supplementary material 2 (DOCX 38 KB) [file 10456_2018_9655_MOESM2_ESM.docx]

Online supplemental material

**Mouse models of Alzheimer’s disease cause rarefaction of pial collaterals and increased severity of ischemic stroke**

Hua Zhang, Bo Jin and James E. Faber

1. Detailed Materials and Methods.

2. References

3. Supplemental Data:

Supplemental figure I. Representative images of collaterals nearing complete pruning (rarefaction) in 3xTg-AD mice.

Supplemental figure II. AD-induced collateral rarefaction (Figure 1) was association with modestly reduced brain and body weight and area of the collateral zone between the MCA-ACA trees. Distal-most arterioles (DMA) of the MCA tree, which are nearby and of similar diameter as collaterals, evidenced no loss of number or diameter.

Supplemental figure III. In contrast to collaterals, AD did not induce loss of number or diameter of PAs branching from the extant collaterals or from branches of the ACA tree.

Supplemental figure IV. Collateral rarefaction coincides with presence of intracerebral HO-1 expression.

**Methods**

AD mice were purchased form the Mutant Mouse Regional Resource Center JAX. 3xTg mice were JAX 34830 [B6;129-*Psen1^tm1Mpm^* Tg (APPSwe,tauP301L)1Lfa/Mmjax]. Wildtype (WT) controls were B6.129SF2/J (JAX, 101045) or B6/J.129SF1/J. 2xTg mice were JAX 34833 [B6.Cg-Tg(APP695)3Dbo Tg(PSEN1dE9)S9Dbo/Mmjax]. Controls were C56BL/6J (B6) WT littermates mice. 1xTg mice were JAX 34843 [C57BL/6.Tg(Thy1-APPSwDutIowa)BWevn/ Mmjax]. WT controls were F1 hybrids of B6 (JAX 00066) mated with C57BL/6NJ (B6N; JAX 005304). B6.CX_3_CR_1_^-/-^ mice were JAX 008451 [B6.129P(Cg)-Ptprc^a^Cx3cr1^tm1Litt^/LittJ]. B6.eNOS overexpressing transgenic mice were described previously.^1^ Mice were fed normal chow and maintained on a standard 12hr light-dark cycle. Approximately equal numbers of both sexes were studied. However, the aims of the study did not include investigation of sexual dimorphism in AD-induced rarefaction, thus n-sizes were not powered to test for sexual dimorphism in this or other endpoints. All procedures were approved by the University of North Carolina’s Institutional Animal Use and Care Committee and the NIH Guide for the Care and Use of Laboratory.

**Angiography and morphometry**

As previously described (see also “detailed protocol…” below),^1-3^ animals were anesthetized deeply with ketamine and xylazine (100 and 15 mg/kg, ip) and heparinized (1000 units/mouse, ip). The aorta at the level of the diaphragm was cannulated, the right atrium perforated, followed by exsanguination with infusion of sodium nitroprusside (10^-4^M, for maximal dilation) in phosphate buffered saline (PBS) containing Evan’s blue dye (provides light staining of brain and abluminal endothelial surface) at 100 mmHg. Yellow Microfil (Flowtech Inc, Carver, MA) was infused at a viscosity adjusted to fill the pial arterial and collateral circulations while minimizing capillary transit and venous filling. Morphometry was conducted as described previously.^4^ After the Microfil had set, brains were kept in 4% PFA. Collaterals were imaged the next day using a Leica fluorescent stereomicroscope. All collaterals between the anterior cerebral artery (ACA) and middle cerebral artery (MCA) trees of both hemispheres were counted; images were then saved into Image J (NIH) for subsequent analysis: Lumen diameter and tortuosity of all collaterals was determined at 50X and averaged for each mouse. Diameter was determined at midpoint. The beginning- and end-points of the collateral were defined as the points of departure of the collateral from the ends of the parent distal-most arteriole of the MCA and ACA tree. Collateral tortuosity was calculated as the vector length/axial length between the two points. Number of penetrating arterioles (PAs) branching from the dorsally imaged ACA tree and their lumen diameter (determined at a distance equal to one diameter of the parent vessel away from the PA’s branch point) were determined from brains optically cleared using methyl salicylate.^5^

**Permanent middle cerebral artery occlusion (pMCAO) and determination of infarct volume**

As previously described,^2-4^ mice were anesthetized with ketamine and xylazine (100 and 10mg/kg, ip, respectively) and rectal temperature was maintained at 37±0.5°C. The temporalis muscle between the eye and ear on one side was retracted after a 4mm incision. After a 2mm craniotomy (18000-17 drill, FST, Foster City, CA), the dura was incised with a 27 gauge needle tip and reflected to reveal the main trunk of the MCA which was cauterized (18010-00, FST, modified) distal to the lenticulostriate branches. The incision was closed with suture and Vetbond (3M, Minneapolis, MN), intramuscular cefazolin 50mg/kg and buprenorphine were administered, and the animal was monitored in a warmed cage during recovery from anesthesia to maintain the above rectal temperature. Mice were euthanized 24h later. Brains were removed and sliced into 1 mm coronal sections that were incubated in 2% 2,3,5-triphenyltetrazolium chloride in PBS at 37°C. Total forebrain and infarct areas were imaged with a stereomicroscope and analyzed with ImageJ (NIH). Percent infarct volume was normalized to forebrain volume.

**Immunohistochemistry**

Whole brains and dissected regions were cryoprotected and embedded in OCT and kept frozen. 8-10 um sections were cut for IHC staining. Sections were treated with PBS with 0.3% Triton-100 for 1hr at room temperature and then blocked with 10% normal donkey serum. Heat-induced epitope retrieval was used for 8-OHdG staining in a Decloaking Chamber (DC2012, Biocare Medical, Concord CA, USA), followed by incubation with 1:200 rat polyclonal anti-CD11b antibody (Ab64347, Abcam, Cambridge MA, USA), 1:200 rabbit anti-NF-kB (Ab16502, Abcam, MA, USA), rabbit anti-HO-1 (Ab13243), 1:150 rabbit anti-SOD2 (Ab13533), 1:100 goat anti-8-OHdG (Ab10802), 1:250 rabbit anti-mouse p16INK4a (Ab189034) 1:50 phospho-eNOS (sc-12972, Ser 1177, Santa Cruz Biotech, Dallas TX), 1:50 eNOS (sc-654), 1:200 secondary antibodies conjugated with Alexa fluor® 568 (A10042, ThermoFisher Scientific, Grand Island, NY, USA) or Alexa fluor® 488 (A21208). For staining for beta-amyloid (Aβ), brain tissues or sections were treated with 70% formic acid for 30 min at RT. After rinsing with water briefly, tissues or sections were treated with 0.3% Triton X100 in PBS for 20 min. Then biotinylated Aβ 6E10 monoclonal antibody (Covance, SIG-39340) was applied at 1;100 dilution and incubated at 4ºC overnight. After washing with PBS 3 times, Alexa Fluor® 488 streptavidin (S11223, Thermo Fisher Scientific) was applied. Images were taken with a Zeiss 880 confocal microscope. Signal intensity was measured with ZEN2012 software (Blue edition, Carl Zeiss Microsope GmbH, 2011). Quantification was as follows: ROIs were manually drawn of the target cells or the mural cell layer (ie, all cells encircling the endothelial cells) of a selected collateral with the Graphics panel of Zen2012. For collateral endothelial cells, the ROI consisted of the isolectin-labeled layer. ZEN2012 automatically measured the fluorescent intensity in the ROI at each of the different wavelength’s channel when different antibodies were used to co-labeled the same cell type or structure, ie, eNOS and isolectin-labeled endothelial cells. Antibodies for CD11b, p16INK4a and 8-OHdG labeled individual cells or nuclei. Antibodies for SOD2 and HO-1 labeled both endothelial and mural cells, thus fluorescent intensity was determined for the entire collateral wall’s ROI.  For Figure 6 and 7 data, based on DAPI, it was not possible to differentiate individual cells versus cell processes for NFkB, SOD2, HO1 and eNOS, unlike for p16INK4a and 8-OHdG; positive points of anti-CD11b staining in the perivascular region of the collateral were counted as individual cells regardless of co-localization with DAPI, since these cells were monocytic-like in appearance. Three different sections of the same collateral were quantified and the results averaged for each mouse.

**Statistics**

Study design and statistical analysis followed best practices in accordance with ARRIVE and STAIR guidelines:^6^ e.g, n-sizes (number of animals) were chosen based on our previous studies which demonstrated sufficient power to test hypotheses about outcomes measured herein; approximately equal number of males and females were included; the investigators were blind to mouse type during data analysis; no data points were identified as outliers and none were excluded; all results were fully disclosed; the review, discussion and citation of the literature was unbiased. pMCAO was used to permanently recruit blood flow across pial collaterals.

Our overarching research hypothesis specified the following:

(1) a causal relationship between collateral rarefaction and subsequent risk of increased infarct volume seen in AD,

(2) a causal relationship between collateral number and diameter and subsequent infarct volume after permanent MCA occlusion,^2,3^

(3) a role for aging and other vascular risk factors which cause directionally similar changes in brain tissue (as evidenced in our previous publications),^1,4,7^ and

(4) that brain tissue of AD mice is well-known to display increased oxidative stress and inflammation relative to wild-type mice.

These components of the overarching hypothesis were addressed in separate experiments. For each experiment, the statistical analyses focused on sample means ± 1 standard error (SE) for estimation of population means. The sample mean ± 1 SE is an approximate 66% confidence interval when n ≈ 10. Tests of null hypotheses (specified *a priori*) relied on one‑sided two-sample Students’ t-tests of size α = 0.05. A two-sided test was used in one experiment (illustrated in Figure 2F) because a one-sided hypothesis and rationale could not be formulated *a priori* based on previous publications. The experiment summarized in Figure 8 used WT and 2xTg-AD mice (from the experiment summarized in Figure 1) along with comparable mice featuring either the eNOS^Tg^ allele or the CX_3_CR1^-/-^ knock out. The primary comparison of interest was of AD-model mice with and without the eNOS^Tg^ allele, or of AD-model mice with and without the CX_3_CR1^-/-^ knock out. Analysis of these comparisons relied on age-specific sample means ± 1 SE, along with one-sided two-sample t-tests of size α = 0.05.

**Detailed protocol for filling the arterial circulation for visualization of pial collaterals**

Preparation of Microfil working solution: each mouse needs ~1 ml: Add 800 ul yellow Microfil and 100 ul diluent to an Eppendorf tube and vortex. When ready to fill the brain, add 100 ul curing agent, vortex and infuse per below. Based on our measurements of viscosity in glass capillary tubes, yellow Microfil and the catalyst remain stable (shelf life) for 12 months for use for pial angiography as done herein.

Preparation of Evans blue dye PBS solution: Make 50 mls of 50 mg/ml Evans blue in PBS in a 50-ml tube. Shake for 4~5 hr. Filter the solution through 0.4 uM filter with vacuum tube. This stock can be kept and used for many mice, as ~ 0.2 cc is used for each mouse. Don’t use Evans blue dye if casting striated muscle.

Filling protocol:

- Inject 1000 units heparin ip.
- Inject 0.1 ml/~25g mouse ketamine/xylazine ip.
- When pedal pinch reflex is absent, place mouse in a supine position and expose descending thoracic aorta at the level of the diaphragm. Cannulate the aorta with pulled out PE-50.
- Perfuse with 1x PBS containing nitroprusside (NP, 10^-4^M) for 3 min (~60 ml needed total here and for below) at ~100 mmHg with a reservoir set to 140 cm height. Cover reservoir with foil to protect from light. NP is prepared as 100x stock solution (29.8 mg in 10 ml PBS) fresh and used immediately. It is rapidly degraded by light and room temperature.
- Carefully turn mouse to prone position, open the skull gently without damaging the dura and pia, and image the dorsal cortex under a dissection scope. Make sure the cannula position remains stable. Remove the dura and keep the PBS+NP perfusion running. This assures that the vessels remain maximally dilated; SMCs could contract and prevent complete filling in the next step below. SMC contraction has lower sensitivity to inhibition by hypoxia; temperatures between 14-30C can induce contraction.
- Perfuse with Evans blue PBS from a reservoir (20 cm height) until the pial collateral circulation is filled but the venous vasculature is only partially filled.
- Switch back to PBS+NP and keep the perfusate running slowly.
- Pour the 1 ml of mixed Microfil into a 1 ml syringe. After expelling air bubbles, connect blunt ended needle to the PE catheter, making sure to not introduce air.
- Inject Microfil into the pial vasculature slowly while watching under scope. Do not over-inject, vis a vis pressure, or Microfil will cross the capillary bed and fill the venules.

Stop injecting when all of the collaterals are filled and the beginning of a small number of capillaries and an occasional venule begin to fill – assures all collaterals have filled. Temper the syringe pressure and slow the infusion so that filling does not continue when pushing of the syringe plunger is stopped (because pressure buildup can cause “over-fill” of venules/veins and obscure detection of collaterals). Do not remove the syringe needle from the PE tubing. Evans blue dye is used because it adheres to the ablumenal surface of the vessels. This allows confirmation, in the occasional case where the center-most segment of a given collateral did not completely fill with Microfil, that it is a collateral and not a distal-most arteriole appearing to end bluntly in the pia as it descends into the parenchyma.

- Flood the pial surface immediately with 4% PFA in PBS to fix the SMCs and prevent any vasoconstriction, as the dilatory effects of nitroprusside are lost with time. This could cause an inaccurate measurement of number or diameter of the collaterals (or other pial vessels). Topical PFA also washes away Microfil that may have leaked out of any broken vessels or bone edges. Cover the brain with a kimwipe, wet it with PFA to prevent drying, and allow Microfil to cure ~20 min.
- Remove head and fix in 4% PFA for >2h (usually at least overnight). Brain can then be removed if desired.

Microfil can be purchased at <http://www.flowtech-inc.com/orderform.asp>. Yellow Microfil contains lead and chromium sulfates (**precaution, toxic**). It is therefore x-ray opaque and may be imaged with a Faxitron or microCT, although x-ray and most microCT instruments do not have the resolution to image vessels less than 15-30 microns in diameter. Other colors without these metals are available, but we have found blue Microfil is not optimal. The whole brain can be counterstained to aid contrast with the pial vasculature, including with TTC staining (however, do not post-fix the brain/head, as above): After the Microfil has cured, place brain in 30 ml of 1% TTC for 30 min.

Tissue clearing protocol:

Various methods can be used to optically clear the brain and other tissues that have been filled with Microfil. See the above manufacturer’s website for some of them, reference 5 below, and other methods recently described for clearing tissues.

We used the following protocol for clearing brain in the present study.

- After the filling with Microfil, brain is fixed in 2% PFA for 24 h.
- Place brain in 15 ml polypropylene tube and add 12 ml 25% ethanol (in water), then place the tube in rocking shaker for 24h.
- Change to 50%, 75%, 95% ethanol and in rocking shaker for 24 h for each percent.
- Blot brain on a paper towel and place in new 15 ml tube; add 12 ml 100% ethanol, rocking-shaker for 24h.
- Blot brain on a paper towel and place in a new 15 ml tube; add 12 ml methyl salicylate (M6752, Sigma), rocking-shaker for 24h. Brain tissue now cleared for viewing. The above clearing protocol causes yellow Microfil to shrink by 65%, which is then used as a correction factor to correct imaged lumen diameters back to anatomic diameters.

**References**

1. Rzechorzek W, Zhang H, Buckley BK, Hua H, Pomp D, Faber JE. Exercise training prevents rarefaction of pial collaterals and increased severity of stroke with aging. J Cere Blood Flow Metab. 2017;37:3544-3555.

2. Lucitti JL, Sealock R, Buckley BK, Zhang H, Xiao L, Dudley AC, Faber JE. Variants of Rab GTPase-effector binding protein-2 cause variation in the collateral circulation and severity of stroke. Stroke. 2016;47:3022-3031.

3. Zhang H, Prabhakar P, Sealock R, Faber JE. Wide genetic variation in the native pial collateral circulation is a major determinant of variation in severity of stroke. J Cerebral Blood Flow Metab. 2010;30:923-934.

4. Faber JE, Zhang H, Lassance-Soares RM, Prabhakar P, Najafi AH, Burnett MS, Epstein SE. Aging causes collateral rarefaction and increased severity of ischemic injury in multiple tissues. Arterioscler Thromb Vasc Biol. 2011;31:1748-1756.

5. Zhang H, Faber JE. [De-novo collateral formation following acute myocardial infarction: Dependence on CCR2+ bone marrow cells.](http://www.ncbi.nlm.nih.gov/pubmed/26254180) J Molec Cell Cardiol. 2015;87:4-16.

6. RIGOR. Improving the quality of NINDS-supported preclinical and clinical research through rigorous study design and transparent reporting. 2012. <http://www.ninds.nih.gov/funding/transparency_in_reporting_guidance.pdf>

7. Moore SM, Zhang H, Maeda N, Doerschuk, Faber JE. [Cardiovascular risk factors cause premature rarefaction of the collateral circulation](http://scholar.google.com/scholar?cluster=10364601404564528447&hl=en&oi=scholarr) and greater ischemic tissue injury. Angiogenesis. 2015;18:265-281.
